# Supplementary material for: Designed 2D protein crystals as dynamic molecular gatekeepers for a solid-state device
Source: Nat Commun. 2024 Jul 27;15:6326. doi: 10.1038/s41467-024-50567-8 (PMC11283500; doi:10.1038/s41467-024-50567-8)
Supplement: Supplementary file 1 — Supplementary Information [file 41467_2024_50567_MOESM1_ESM.pdf]

Supplementary Information

for

Designed 2D Protein Crystals as Dynamic Molecular Gatekeepers for a Solid-State Device

Sanahan Vijayakumar*[1], Robert G. Alberstein*[2], Zhiyin Zhang*[2], Yi-Sheng Lu[1], Adriano Chan[3], Charlotte E. Wahl[4], James S. Ha[4,5], Deborah E. Hunka[4], Gerry R. Boss[3], Michael J. Sailor[#,1,2,6], F. Akif Tezcan[#,1,2]

[1]Materials Science and Engineering Program, University of California, San Diego, La Jolla, California, 92093, United States

[2]Department of Chemistry & Biochemistry, University of California, San Diego, La Jolla, California, 92093, United States

[3]Department of Medicine, University of California, San Diego, La Jolla, California, 92093, United States

[4]Leidos, 4161 Campus Point Ct, San Diego, California, 92121, United States

[5]Current address: Battelle, 505 King Ave Columbus, Ohio, 43201, United States

[6]Department of Nanoengineering, University of California, San Diego, La Jolla, California, 92093, United States

[*]These authors contributed equally

[#]Corresponding author: tezcan@ucsd.edu, msailor@ucsd.edu

Table of Contents

Supplementary Figure 1. White light reflectance measurements of MCbi-dye-impregnated pSi photonic crystal sensors without a ^{CEE}RhuA gatekeeper in response to air saturated with hexane vapor.

Supplementary Figure 2. Spectroscopic characterization and surface morphology of photonic crystal sensors.

Supplementary Figure 3. Series of optical absorbance spectra of MCbi with sequential addition of cyanide ion.

Supplementary Figure 4. Molecular dynamics simulations of hexane permeation through single-layer RhuA lattices.

Supplementary Figure 5. Temporal responsive curve of MCbi-dye-impregnated pSi photonic crystal sensors with a ^{CEE}RhuA gatekeeper in response to HCN and purged air.

Supplementary Figure 6. Spectroscopic measurements of pSi sensors coated with a ^{CEE}RhuA gatekeeper in response to hexane and NH_3.

Supplementary Figure 7. Spectroscopic measurements of Co^{2+}-free (ajar) ^{CEE}RhuA-coated pSi sensors in response to hexane and HCN vapors, illustrating the challenge of detecting cyanide in the presence of a common interferent.

Supplementary Figure 8. Spectroscopic measurements of pSi sensors coated with disassembled ^{CEE}RhuA crystals and Co^{2+} in response to hexane and HCN vapors.

Supplementary Figure 9. Spectroscopic measurements of pSi sensors coated with non-chemically responsive $^{\text{C98}}$RhuA crystals and Co^{2+} in response to hexane and HCN vapors.

Supplementary Figure 10. Negative-stain TEM images of ^{CEE}RhuA gatekeeper in response to TCEP.

Supplementary Figure 11. Spectroscopic measurements of pSi sensors coated with a ^{CEE}RhuA gatekeeper in response to ethanol.

Supplementary Figure 12. Negative-stain TEM images of solution-state ^{CEE}RhuA gatekeeper in response to ethanol.

Supplementary Table 1. Summary of existing HCN sensors.

Supplementary Table 2. Summary of gatekeeper controls and conditions.

Supplementary Table 3. Statistical Significance of Gatekeepers in Figure 4b.

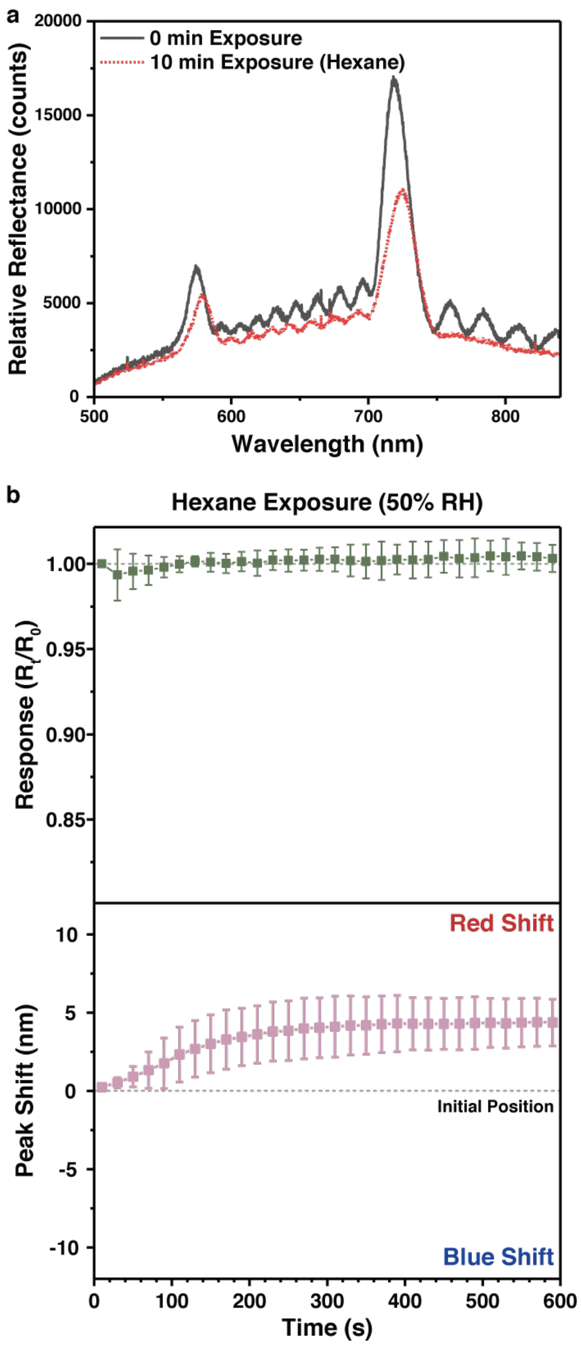

Supplementary Fig. 1. White light reflectance measurements of MCbi-dye-impregnated pSi photonic crystal sensors without a ^{CEE}RhuA gatekeeper in response to air saturated with hexane vapor. a. Reflectance spectra of pSi sensor obtained before and after a 10-minute exposure to air (50% RH) saturated with hexane vapor (3100 ppm). The lack of the ^{CEE}RhuA gatekeeper enables hexane to penetrate the pores of the sensor, shifting the stop bands to the red, and decreasing their intensity. **b.** The change in normalized signal response (I_{signal} / $I_{reference}$) and the shift in the wavelength of the peak maximum are shown upon exposure to the hexane vapor. Error bars represent the standard deviation obtained from measurements done in triplicate.

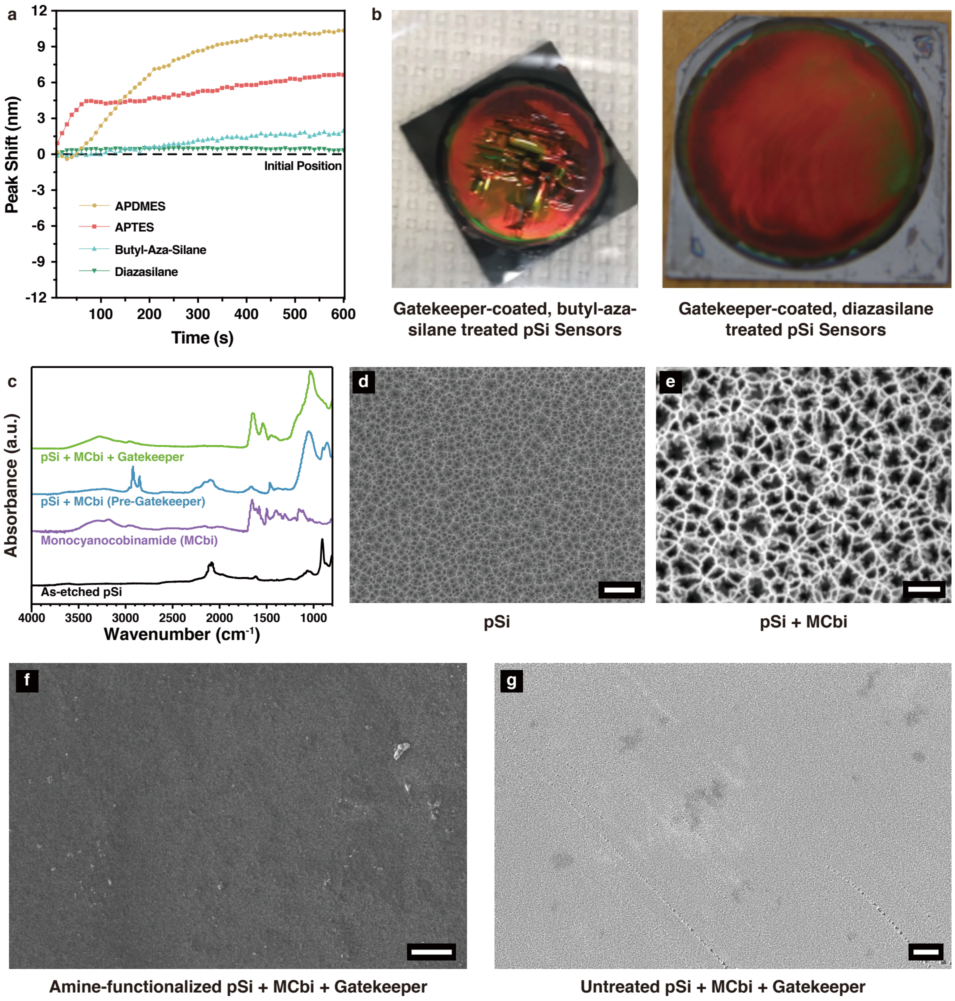

Supplementary Fig. 2. Spectroscopic characterization and surface morphology of photonic crystal sensors. a. Temporal wavelength peak maximum shift response of gatekeeper-coated pSi photonic crystals functionalized with various aminosilanes upon exposure to 3100 ppm hexane vapor. As hexane infiltration into the surface pores of the pSi substrate leads to redshifting of the reflectance peaks (**Supplementary Fig. 1**), peak shift was used as an indicator for effective coverage of the pSi sensor with gatekeeper crystals. Each amine-functionalized silane was passivated onto as-etched pSi wafers and subsequently coated with the gatekeeper. Shown here are the alkoxysilanes APDMES (3-aminopropyl(dimethyl)ethoxysilane) and APTES ((3-aminopropyl)triethoxysilane) and the heterocyclic azasilanes "butyl-aza-silane" (N-n-butyl-aza-2,2-dimethoxy-silacyclopentane) and "diazasilane" (2,2-dimethoxy-1,6-diaza-2-silacyclooctane)). The heterocyclic azasilanes enabled a consistent aminated pSi surface with high coupling efficiency for gatekeeper deposition, without pSi pore clogging, a consequence of commonly used alkoxysilanes. The heterocyclic-azasilane-aminated pSi photonic crystals proved to be effective gatekeeper attachment surfaces, as attributed to the lack of peak-shift after hexane exposure, with "diazasilane" showing the smallest shift. **b.** Photographs of heterocyclic-azasilane-treated pSi photonic crystals 48 h after gatekeeper deposition. To ensure long-term robustness of the sensor, diazasilane was selected for final sensor preparation as "butyl-aza-silane"-treated pSi resulted in visible flaking of the gatekeeper-coated porous thin film after two days in ambient conditions. **c.** Attenuated total reflectance Fourier-transform infrared (ATR-FTIR) spectra of the photonic crystal sensor and its components, from bottom to top: As-etched pSi (black trace); monocyano-cobinamide (MCbi) (purple trace); the pSi sample after amination with diazasilane and deposition of MCbi (pSi + MCbi, blue trace) and the pSi sample after deposition of MCbi followed by coating with the gatekeeper (pSi + MCbi + Gatekeeper, green trace). Representative plan-view scanning electron microscope (SEM) images of **d.** as-etched porous Si photonic crystal (scale bar = 5 μm), **e.** MCbi-impregnated pSi photonic crystal (scale bar = 100 nm), **f.** Gatekeeper-coated, MCbi-impregnated, diazasilane-functionalized pSi photonic crystal (scale bar = 500 nm), and **g.**

Gatekeeper-coated, MCbi-impreganted, bare pSi photonic crystal (scale bar = 1 μm). The micrograph in **f** was selected with a small amount of pSi substrate visible to depict how the addition of the gatekeeper coating covers the mesoporous features of the porous silicon substrate. The micrograph in **g** demonstrates the necessity of surface amination for gatekeeper adhesion, as the deposited crystals are not observed after the wash step, while they are clearly visible in **f**.

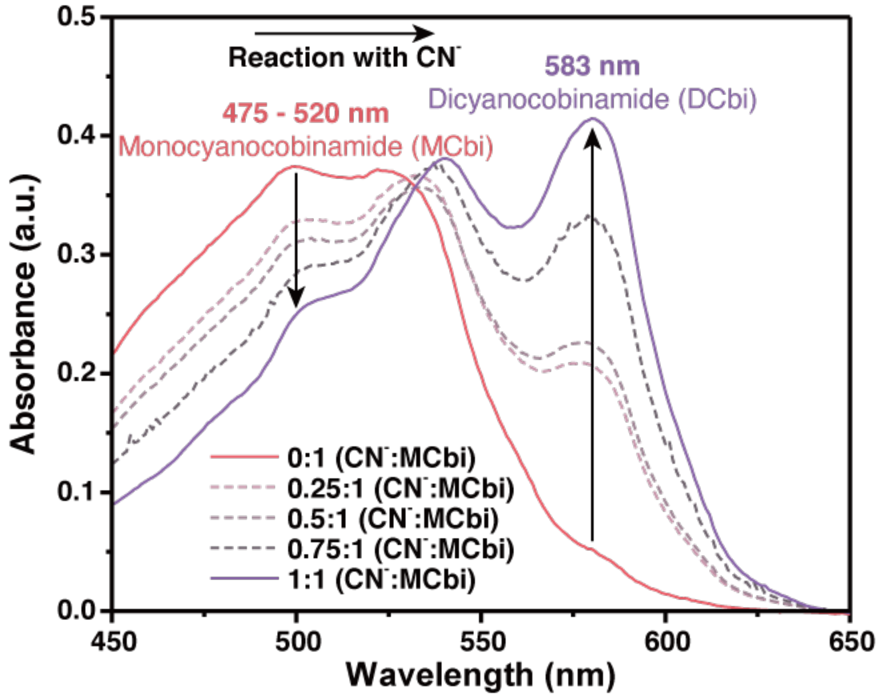

Supplementary Fig. 3. Series of optical absorbance spectra of MCbi with sequential addition of cyanide ion. Series of UV-vis absorbance spectra of monocyano-cobinamide (MCbi) (500 μM) in water (red trace), upon titration with various ratiometric concentrations of CN^-, as indicated in the legend, to yield the fully CN-complexed dicyano-cobinamide compound (DCbi, purple trace). Monocyano-cobinamide (“$CN(H_2O)Cbi$”, or “MCbi”) was used and prepared in-situ by reaction of an equimolar mixture of aquohydroxocobinamide and KCN.

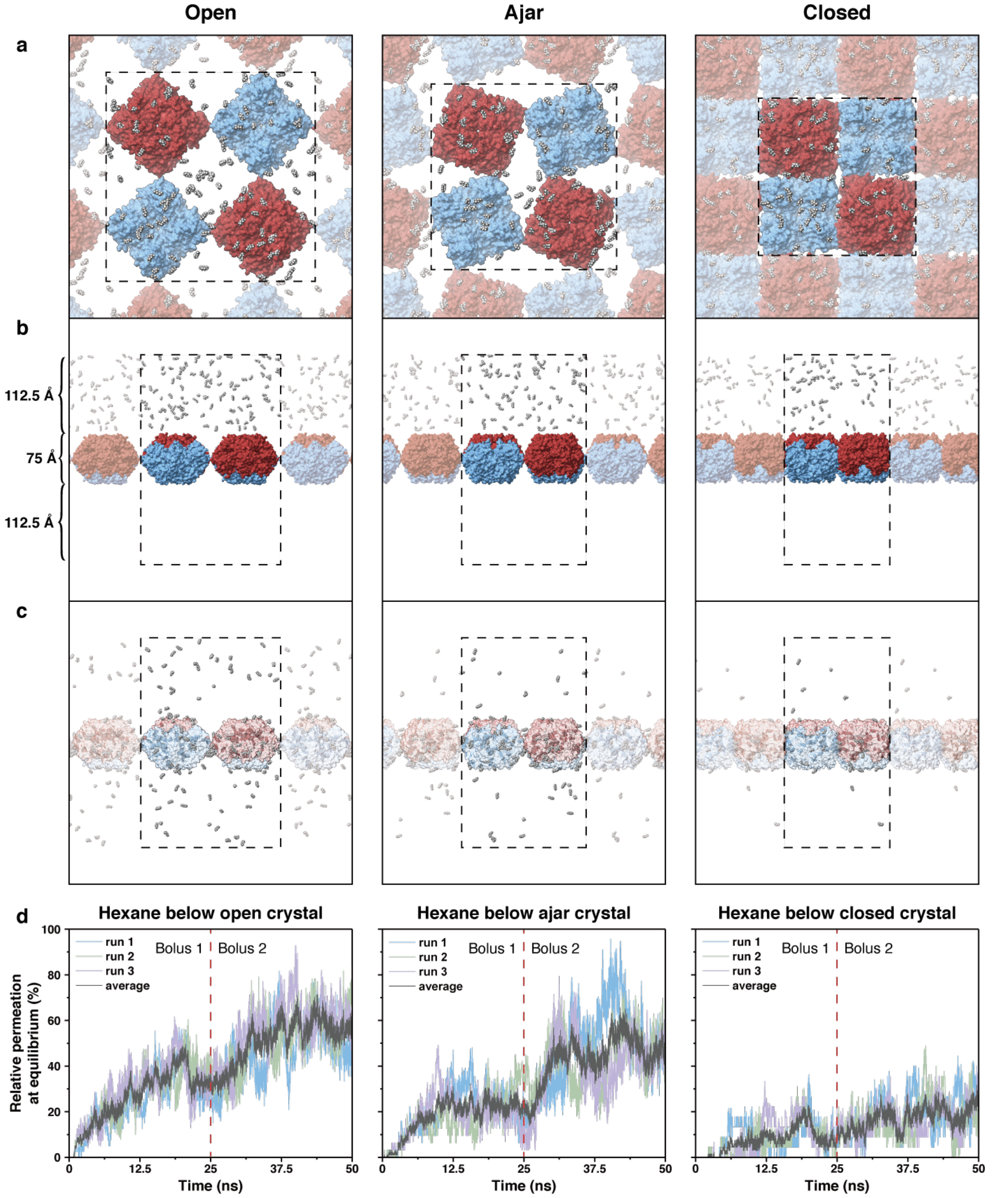

Supplementary Fig. 4. Molecular dynamics simulations of hexane permeation through single-layer RhuA lattices. a. Top-down and **b.** side views of the initial state of infinitely periodic simulations of individual ^{C98}RhuA crystals in the three relevant conformations. The simulations were carried out *in vacuo* with a total height of 300 Å (central box bounded by dashed lines), with equilibrated gas-phase neat hexane (3130 ppm) placed above the layer. **c**. Final frame of each 50 ns simulation, highlighting the number of hexanes below the crystal (*i.e.*, within the pSi sensor) as well as the large number of adsorbed hexane molecules on the protein surfaces. **d.** Number of hexane molecules below the lattice over time for three independent replicate simulations, normalized to uniform density, showing similar permeability for open and ajar states that are both greater than the closed crystal. At 25 ns, a second bolus of neat hexane was added to the top of the simulation to approximate the conditions of continuous flow experienced in the experimental apparatus. Note that only the ajar and closed states are experimentally relevant; the fully open state was computed as a reference point, demonstrating that the permeability of ajar-state crystals is comparable to the maximally porous configuration while closed-state crystals are distinct.

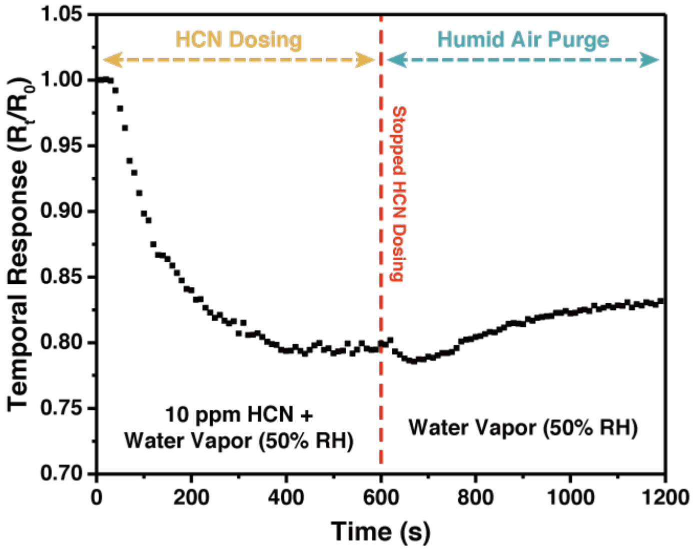

Supplementary Figure 5. Temporal response curve of MCbi-dye-impregnated pSi photonic crystal sensors with a ^{CEE}RhuA gatekeeper in response to HCN and purged air. Representative temporal responsive curve of gatekeeper-coated photonic crystal sensor to a sequential exposure of 10 ppm HCN in 50 % RH air (time = 0-600 s) followed by a 50 %RH purge (time = 600-1200 s). The optical responses were obtained every 10 s for both conditions.

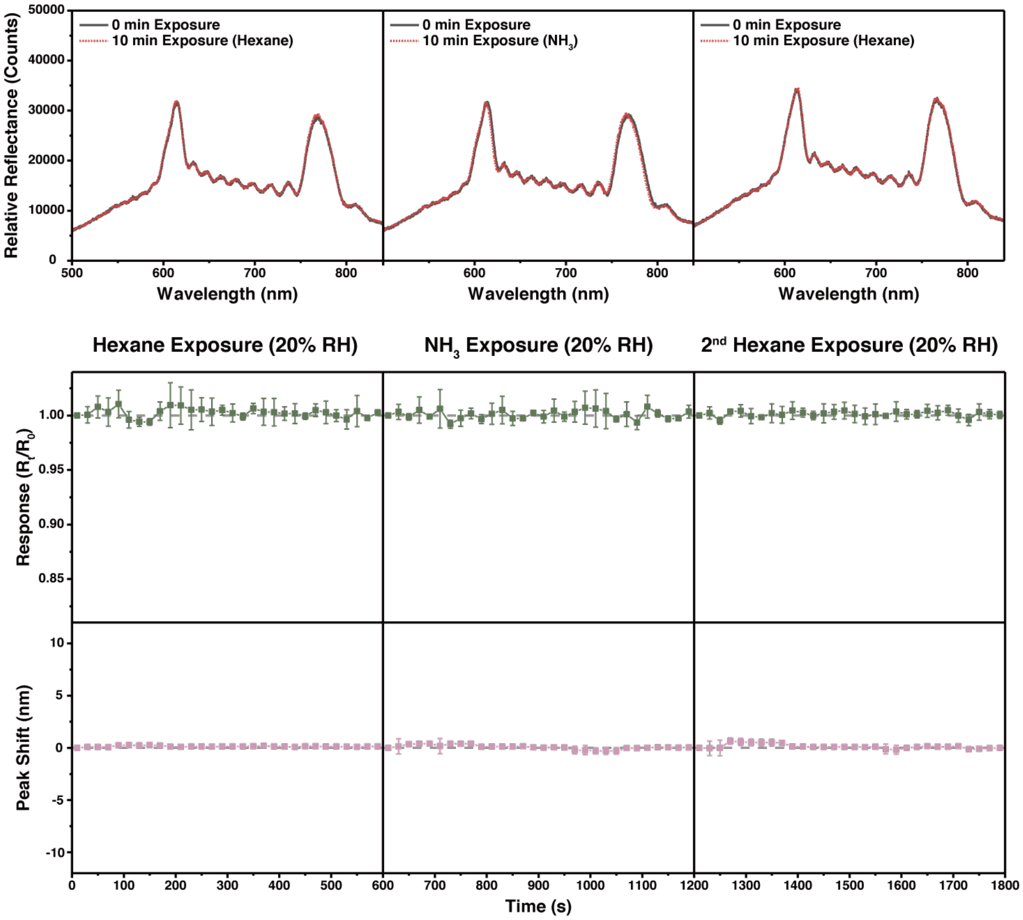

Supplementary Fig. 6. Spectroscopic measurements of pSi sensors coated with a CEERhuA gatekeeper in response to hexane and NH_3. Top: White light reflectance spectra of Co-bound-gatekeeper-coated pSi sensors, showing total stability of the spectra before and after sequential exposure of hexane, ammonia, hexane. Bottom: the Response panels correspond to those seen in the analogous experiment (using HCN instead of NH_3) in **Figure 3c**, and the Peak Shift panels depict the actual movement of the reflectance spectra that occurs upon interferent infiltration (as seen in **Supplementary Fig. 1**). Notably, the use of NH_3 as a substitute for HCN does not produce any peak shift in the second hexane exposure, demonstrating that the gatekeeper remains firmly closed, in contrast with the data in **Figure 3c** for HCN. Error bars represent the standard deviation obtained from measurements done in triplicate.

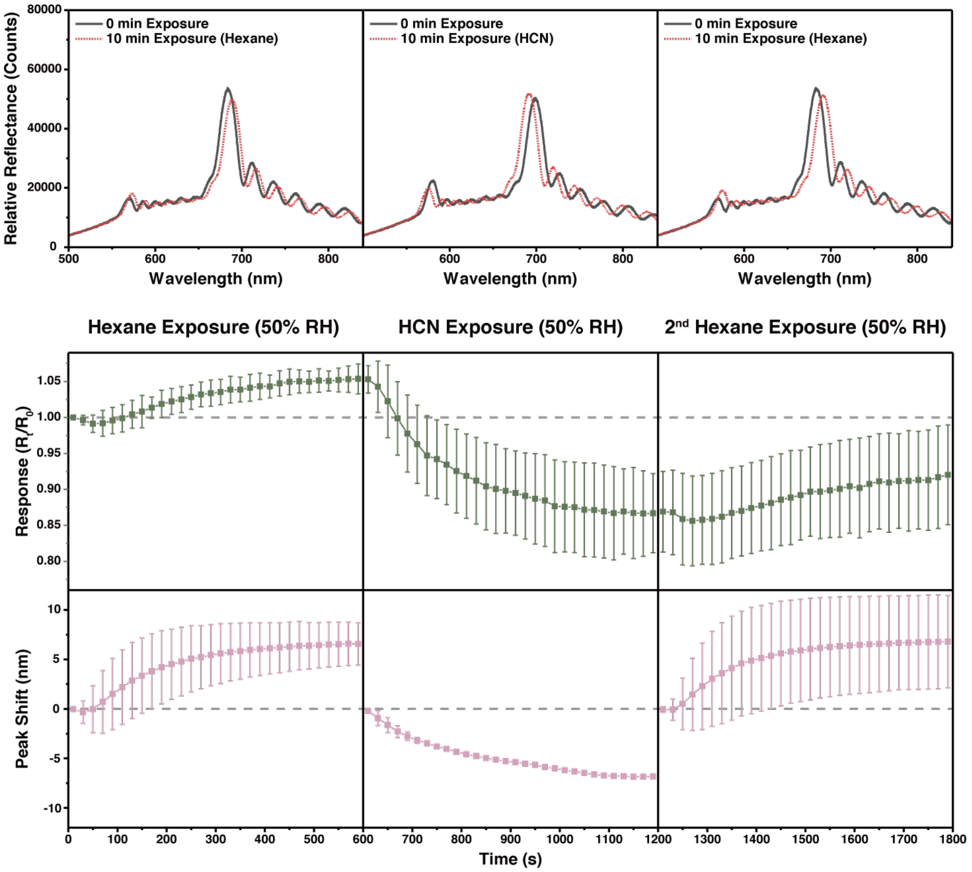

Supplementary Fig. 7. Spectroscopic measurements of Co^{2+}-free (ajar) ^{CEE}RhuA-coated pSi sensors in response to hexane and HCN vapors, illustrating the challenge of detecting cyanide in the presence of a common interferent. The Co^{2+}-free, ^{CEE}RhuA-coated sensor corresponds to the gatekeeper locked in its "ajar" state and does not act as a cyanide-responsive gatekeeper. **Top panels.** White light reflectance spectra (uncorrected for instrument spectral response) of the Co^{2+}-free, ^{CEE}RhuA-coated sensors. The red shift of the two stop bands of the sensor (at ~575 and ~680 nm) after 10 min of exposure to hexane-saturated air is attributed to non-specific adsorption of hexane within the mesoporous silicon matrix, which results in an increase in the average refractive index of the layer. The observed intensity of the stop bands also changes, due to a combination of the change in index contrast in the photonic crystal and the non-linearity of the spectral response of the spectrometer detector. After 10 min of exposure to HCN in flowing air (center) the adsorbed hexane has evaporated, causing the stop bands to shift back to the blue. Concurrent with this shift is a decrease in the relative intensity of the ~575 nm stop band in the photonic crystal due to the strong optical absorbance of CN-MCbi in that region of the spectrum (see **Supplementary Fig. 3**). Exposure to an additional dose of hexane-saturated air (third panel from the left) again results in a red shift of the stop bands and changes in relative intensity due to adsorption of hexane within the mesoporous silicon layer. **Bottom panels.** Changes in the normalized reflected intensity (relative to 0 s) and wavelength of the stop bands (relative to the start of each individual gas stream) are quantified as a function of time for each of the three 10-min experiments given in the top panels. The normalized Response function (I_{signal} / $I_{reference}$), which is the ratio of the intensity of the stop band measured at ~575 nm to the intensity of the stop band measured at ~680 nm, is expected to decrease upon exposure to CN^-, as it does in the hexane-free HCN exposure experiment of the center panel. However, exposure to hexane-saturated air either before (left panel) or after (right panel) exposure to cyanide causes an increase in this Response function, illustrating how a background interferent at high concentration (hexane, in this case) can obscure the HCN response of the sensor. These Co^{2+}-free, ^{CEE}RhuA set of experiments demonstrate the response of the sensor when no HCN-triggered gatekeeper is present. Error bars represent the standard deviation obtained from measurements done in triplicate.

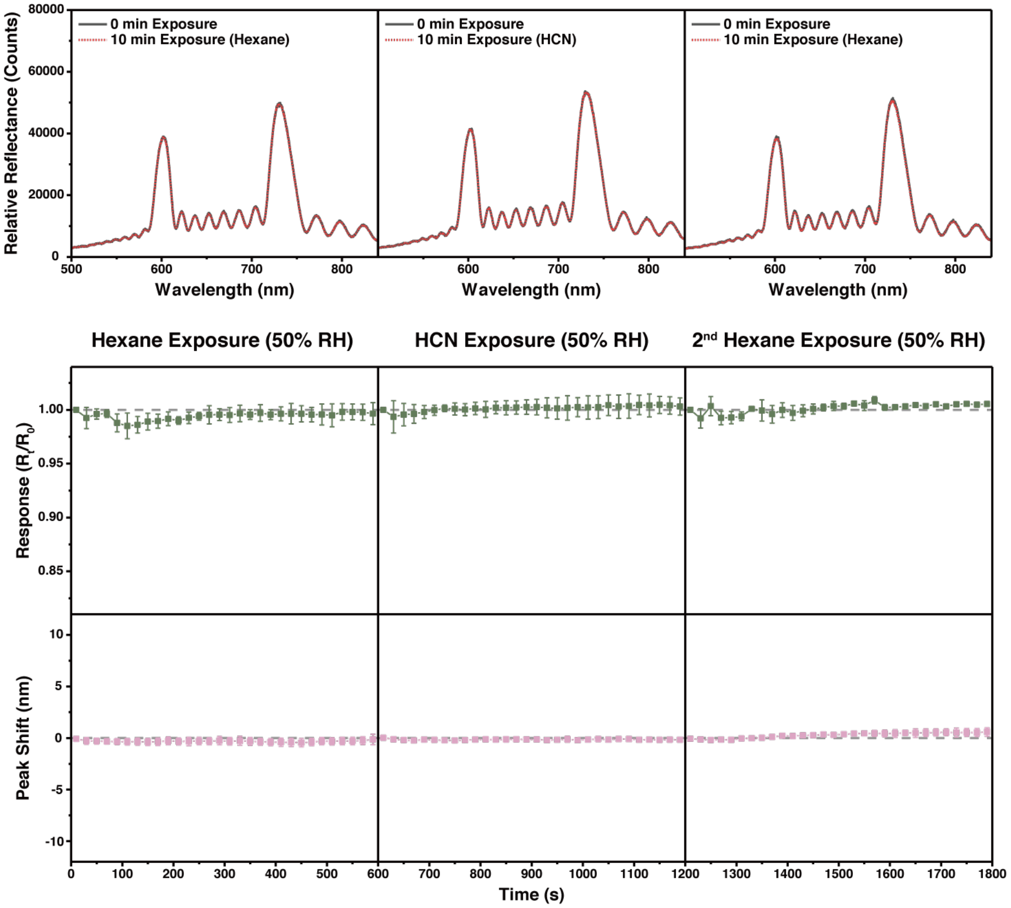

Supplementary Fig. 8. Spectroscopic measurements of pSi sensors coated with disassembled ^{CEE}RhuA crystals and Co^{2+} in response to hexane and HCN vapors. Top and Bottom panels correspond to those in **Supplementary Fig. 6**, wherein changes in the normalized reflected intensity (relative to 0 s) and wavelength of the stop bands (relative to the start of each individual gas stream) are quantified as a function of time for each of the three 10-min experiments given in the top panels, except that the sensor in this case is coated with the disassembled ^{CEE}RhuA protein. The protein was exposed to Co^{2+} for experimental consistency with **Supplementary Fig. 6**, but this protein does not assemble into a 2D structure, does not form channels, and is not responsive to HCN. The lack of HCN- or hexane-dependent changes in signal intensity or peak shifts confirms that the simple protein coating is insufficient to act as a gatekeeper. The protein layer is impermeable to either HCN or hexane, excluding them both from the sensing layer of the pSi photonic crystal. Error bars represent the standard deviation obtained from measurements done in triplicate.

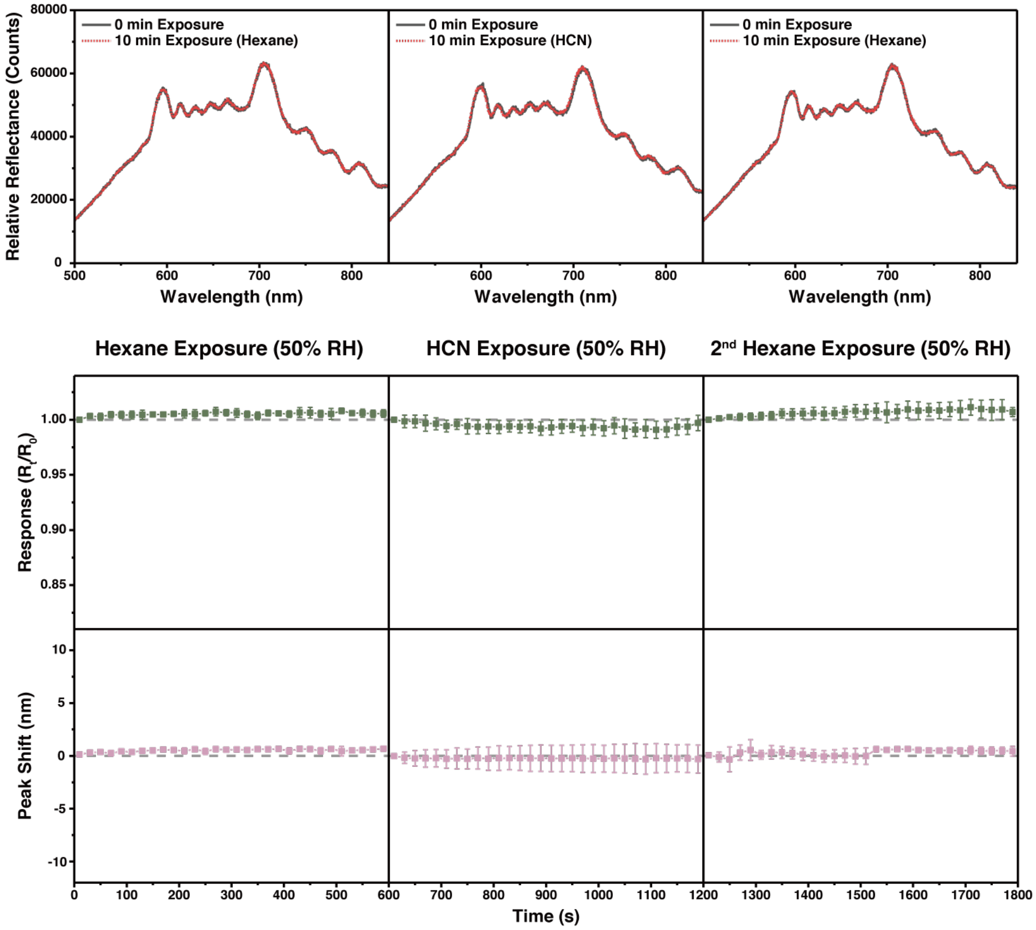

Supplementary Fig. 9. Spectroscopic measurements of pSi sensors coated with non-chemically responsive ^{C98}RhuA crystals and Co^{2+} in response to hexane and HCN vapors. Top and bottom panels correspond to those in **Supplementary Figs. 6** and **7**, except that the protein in this case is the non-chemically responsive ^{C98}RhuA protein. The ^{C98}RhuA protein forms 2D crystals similar to ^{CEE}RhuA but it does not form open channels either with or without addition of HCN—essentially the gatekeeper is locked in a "closed" state. The lack of HCN- or hexane-dependent changes in signal intensity or peak shifts from the pSi photonic crystal sensing layer confirms that the closed gatekeeper blocks compounds from entering the sensing layer. Error bars represent the standard deviation obtained from measurements done in triplicate.

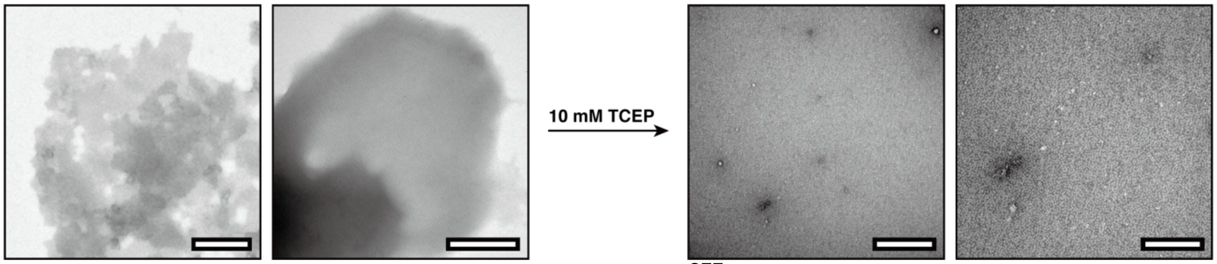

Supplementary Fig. 10. Negative-stain TEM images of ^{CEE}RhuA gatekeeper in response to TCEP. The addition of 10 mM TCEP, a non-sulfurous reducing agent, to assembled ^{CEE}RhuA gatekeeper crystals (left) results in their complete dissolution into RhuA proteins (visible as individual square-shaped units on the right) by breaking the disulfide bonds that hold together the 2D lattices. The scale bars are (from left to right) 500, 200, 500, 200 nm, respectively.

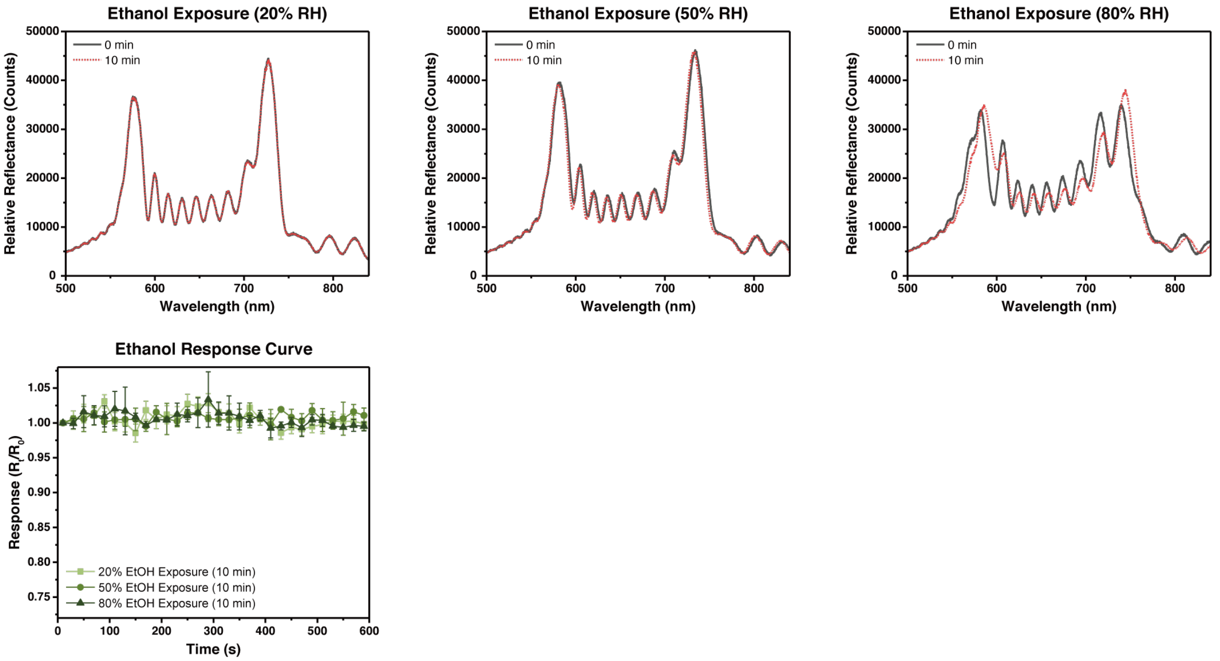

Supplementary Fig. 11. Spectroscopic measurements of pSi sensors coated with a ^{CEE}RhuA gatekeeper in response to ethanol. Top: Reflectance spectra of pSi sensor obtained before and after a 10-min exposure to ethanol at humidity values of 20%, 50%, and 80% RH. At 20% and 50% RH conditions, the sensor shows no noticeable change, however at 80% RH there is a small peak shift implying induced porosity of the layer. Bottom: the lack of change in the normalized signal response (I_{signal} / $I_{reference}$) are shown upon exposure to the ethanol vapor indicating that even with the penetration of amphiphilic interferents, there is no change in the signal response. Error bars represent the standard deviation obtained from measurements done in triplicate.

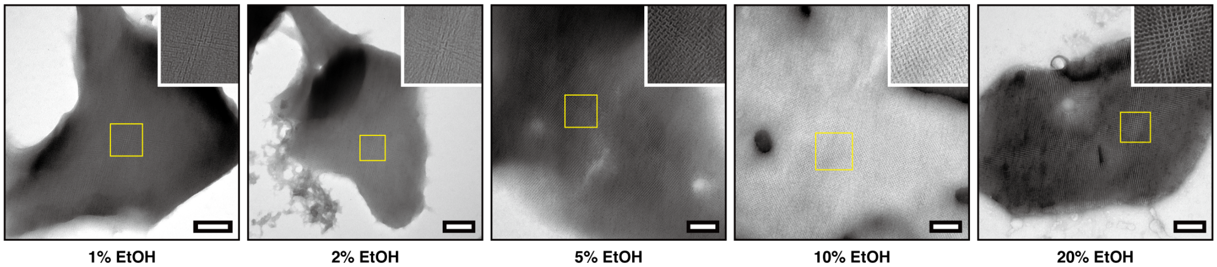

Supplementary Fig. 12. Negative-stain TEM images of solution-state ^{CEE}RhuA gatekeeper in response to ethanol. Negative-stain transmission electron microscope (ns-TEM) images of the resulting ^{CEE}RhuA crystals following exposure to varying concentrations of ethanol. All percentages are reported as v/v. Between 1-10% ethanol, there are no obvious changes in the pore opening, though high molar concentrations of >10% can cause gatekeeper opening and eventually damage to the proteins themselves. It is possible that on the pSi surface, ethanol alone does not negatively affect the gatekeeper, but sufficiently high concentrations of water forming a mixture with the ethanol vapor may result in structural changes to the protein gatekeeper that render it unable to fully reject interferents. The insets are magnified versions of the regions highlighted in yellow to facilitate visualization of the pore conformational state. The scale bars are 100 nm.

Supplementary Table 1. Summary of existing HCN Sensors. List of HCN sensors and their respective sensing modalities.

Reference	Sensing Modality	Sensing Threshold
Hatam, H.; Hanshemi, P. Synthesis of silver nanoparticles-Agarose composite and its application to the optical detection of cyanide ion. Anal. Sci. 2018, 34, 567–570.	Colorimetric Sensor	1.5 to 120 μM
Salahaddin, H.; Khalil, F.; Mehrdad, F.; Reza, E. Sabzi, Silver nanoparticles as a cyanide colorimetric sensor in aqueous media. Anal. Methods 2011, 3, 2599–2603.	Colorimetric Sensor	16.7 to 133.3 μM
Dongtao, L.; Lili, L.; Fengxia, L.; Shaomin, S.; Yingfu, L.; Matin, M.F.C.; Chuan, D. Lysozyme-stabilized gold nanoclusters as a novel fluorescence probe for cyanide recognition. *Spectrochim. Acta Part A* **2014**, *121*, 77–80.	Fluorescence Sensor	5 to 120 μM
Nagamalai, V.; Maria, T.F.A. Novel one-pot and facile room temperature synthesis of gold nanodots and application as highly sensitive and selective probes for cyanide detection. *Nanotechnology* **2016**, *27*, 475505.	Fluorescence Sensor	0.29 to 8.87 μM
Dulal, S.; Samuel, S.R.D.; Anant, K.S.; Tapas, S.; Hongtao, Y.; Paresh, C.R. A Label-Free Gold-Nanoparticle-Based SERS Assay for Direct Cyanide Detection at the Parts-per-Trillion Level. *Chem. Eur. J.* **2011**, *17*, 8445–8451.	SERS Sensor	<1 ppb
Penghui, L.; Pan, L.; Xuecai, T.; Junping, W.; Yunfeng, Z.; Heyou, H.; Liangbao, Y. Assembling PVP-Au NPs as portable chip for sensitive detection of cyanide with surface-enhanced Raman spectroscopy. *Anal. Bioanal. Chem.* **2020**, *412*, 2863–2871.	SERS Sensor	100 ppb
V.G. Marini, E. Torri, L.M. Zimmermann, V.G. Machado<br>An anionic chromogenic chemosensor based on 4-(4-nitrobenzylideneamine)-2,6-diphenylphenol for selective detection of cyanide in acetonitrile-water mixtures<br>Arkivoc, xi (2010), pp. 146-162	Chromogenic Sensor	6 to 600 μM
R. Gotor, A.M. Costero, S. Gil, M. Parra, R. Martínez-Máñez, F. Sancenón, *et al.*<br>Selective and sensitive chromogenic detection of cyanide and HCN in solution and in gas phase<br>Chem. Commun, 49 (2013), pp. 5669-5671	Chromogenic Sensor	1 ppm
Y. Tian, P.K. Dasgupta, S.B. Mahon, J. Ma, M. Brenner, J.-H. Wang, et al.<br>A disposable blood cyanide sensor<br>Anal. Chim. Acta, 768 (2013), pp. 129-135	Optical Absorbance Sensor	0.5 to 200 μM
J. Ma, S.-I. Ohira, S.K. Mishra, M. Puanngam, P.K. Dasgupta, S.B. Mahon, et al.<br>Rapid point of care analyzer for the measurement of cyanide in blood<br>Anal. Chem, 83 (2011), pp. 4319-4324	Optical Absorbance Sensor	≥10 μM
N.B.H. Anh, M. Sharp<br>Determination of cyanide by cathodic stripping voltammetry at a rotating silver disk electrode<br>Anal. Chim. Acta, 405 (2000), pp. 145-152	Electrochemical Detection Sensor	> 1.2 μM
A. Safavi, N. Maleki, H.R. Shahbaazi<br>Indirect determination of cyanide ion and hydrogen cyanide by adsorptive stripping voltammetry at a mercury electrode<br>Anal. Chim. Acta, 503 (2004), pp. 213-221	Electrochemical Detection Sensor	50–800 nM

Supplementary Table 2. Summary of gatekeeper controls and conditions. Table of variations in the protein garekeeper coated onto the photonic crystals. The table shows the various vapor dosing sequences, the expected effect and the observed outcome after vapor dosing. Each variation is attributed to the corresponding figure number detailed in the table.

Gatekeeper – pSi Sample	Vapors Dosed - Sequentially	Expected Effect	Observed Outcome	Figure #
No gatekeeper (pSi + MCbi)	Hexane (50 %RH) – 10 min	Peak Shift and/or change in response	Reflectance peak shift due to hexane	Figure S1
^{CEE}RhuA crystal + $CoCl_2$ (complete gatekeeper)	1. Hexane (20 %RH) – 10 min	Gatekeeper should be fully impermeable to vapers (both hexane and NH_3)	No change in peak shift or response	Figure S6
	1. NH_3 (20 %RH) – 10 min			
	3. Hexane (20 %RH) – 10 min			
^{CEE}RhuA crystal (without $CoCl_2$)	1. Hexane (50 %RH) – 10 min	^{CEE}RhuA crystal without Co^{2+} will be permeable to vapors	Peak shift and response	Figure S7
	2. 10 PPM HCN (50 %RH) – 10 min			
	3. Hexane (50 %RH) – 10 min			
Disassembled ^{CEE}RhuA protein (TCEP-reduced) + $CoCl_2$	1. Hexane (50 %RH) – 10 min	Disassembled ^{CEE}RhuA protein and Co^{2+} will be fully impermeable to all vapors (due to clogging of the pores)	No change in peak shift or response	Figure S8
	2. 10 PPM HCN (50 %RH) – 10 min			
	3. Hexane (50 %RH) – 10 min			
^{C98}RhuA crystal + $CoCl_2$	1. Hexane (50 %RH) – 10 min	Non-responsive ^{C98}RhuA crystal and Co^{2+} should be fully impermeable to vapors	No change in peak shift or response	Figure S9
	2. 10 PPM HCN (50 %RH) – 10 min			
	3. Hexane (50 %RH) – 10 min			
^{CEE}RhuA crystal + $CoCl_2$ (complete gatekeeper)	1. Hexane (50 %RH) – 10 min	Gatekeeper should be impermeable to clutter vapors but show a response to HCN	Gatekeeper opens with HCN exposure	Figure 3
	2. 10 PPM HCN (50 %RH) – 10 min			
	3. Hexane (50 %RH) – 10 min			

Supplementary Table 3. Statistical Significance of Gatekeepers in Figure 4b. Tukey's multiple comparisons test used for the response to the hexane and HCN challenges in Figure 4b. For the hexane challenge, the reported p-value is relative to the Co^{2+}-Free Gatekeeper while for the HCN challenge, the reported p-value is relative to the ^{CEE}RhuA Gatekeeper (*$p < 0.05$, **$p < 0.01$, ***$p < 0.001$, ****$p < 0.0001$; n.s., not significant).

Tukey's Multiple Comparison Test	Challenge	P Value	Summary
Co^{2+}-Free Gatekeeper vs. Disassembled Gatekeeper	Hexane	0.0085	**
Co^{2+}-Free Gatekeeper vs. ^{C98}RhuA Gatekeeper	Hexane	0.045	*
Co^{2+}-Free Gatekeeper vs. ^{CEE}RhuA Gatekeeper	Hexane	0.023	*
^{CEE}RhuA Gatekeeper vs. Disassembled Gatekeeper	HCN	7.8×10^{-6}	****
^{CEE}RhuA Gatekeeper vs. ^{C98}RhuA Gatekeeper	HCN	9.2×10^{-6}	****
^{CEE}RhuA Gatekeeper vs. Co^{2+}-Free Gatekeeper	HCN	0.067	ns
